# Fifteen Years of Orthopedic Malpractice Litigation in Türkiye: A Supreme Court Analysis and International Comparison

**DOI:** 10.3390/jcm15020625

**Published:** 2026-01-13

**Authors:** Uğur Özdemir, Abdülhalim Akar, Muhammed Fatih Serttaş, Aykut Başer

**Affiliations:** 1 Department of Orthopedics and Traumatology, Faculty of Medicine, Bandırma Onyedi Eylül University, 10250 Bandırma, Türkiye; ugurozdemir.md@gmail.com; 2 Bosphorus Spine Center, 34100 Istanbul, Türkiye; ahalimakar@gmail.com; 3 Department of Orthopedics and Traumatology, Kocaeli City Hospital, 41100 Kocaeli, Türkiye; mfserttas@gmail.com; 4 Department of Urology, Faculty of Medicine, Bandırma Onyedi Eylül University, 10250 Bandırma, Türkiye

**Keywords:** orthopedic surgery, malpractice, supreme court, litigation, negligence, trauma

## Abstract

**Background/Objectives**: Orthopedic surgery is among the most frequently litigated medical specialties worldwide. However, high-court malpractice decisions involving orthopedic specialists in Türkiye remain underexplored. This study aims to identify the patterns, causes, and outcomes of malpractice cases involving orthopedists by analyzing Turkish Supreme Court decisions over the past 15 years. **Methods**: A retrospective review of orthopedic malpractice cases adjudicated by the Turkish Court of Cassation between January 2010 and November 2025 was conducted. Variables included type of alleged offense, clinical context, primary/secondary liability, initial court outcomes, high-court decisions, and fault attribution. Findings were compared with international literature to contextualize national patterns. **Results**: A total of 71 decisions were analyzed. Negligent injury was the most common allegation. Initial acquittal and conviction rates were 50.7% and 49.3%, respectively. The Supreme Court affirmed 53.5% of decisions and overturned 46.5%. Fault was attributed to orthopedic specialists in 29.6% of cases, while 40.8% were found faultless; the remaining cases required additional expert evaluation. Litigation themes included diagnostic delay, postoperative complications, inadequate monitoring, and documentation deficiencies. Comparative analysis revealed substantial alignment between Turkish and international malpractice patterns. **Conclusions**: Orthopedic malpractice litigation in Türkiye mirrors global trends, with most claims stemming from trauma-related care and diagnostic errors. Although many cases undergo prolonged appeals, ultimate conviction rates remain low. Strengthened documentation, improved communication, and enhanced clinical guideline adherence may reduce litigation risk and improve patient safety.

## 1. Introduction

Medical malpractice litigation has become a growing global concern in recent decades, affecting clinicians, healthcare systems, and patient–physician relationships across many countries. Orthopedic surgery—due to the invasive nature of procedures, high patient expectations, and the complexity of trauma care—has consistently been identified as one of the most litigated medical specialties worldwide [1,2,3]. Large national datasets and claims-based analyses (e.g., NHS England) show that orthopedic litigation accounts for substantial volume and cost, and frequently clusters around diagnostic, perioperative, and management-related issues [2,4]. Studies from Italy, France, China, and the United Kingdom report that orthopedic surgeons face disproportionately higher malpractice risks compared with other surgical fields, with common allegations including diagnostic delay, perioperative complications, surgical errors, inadequate monitoring, and insufficient informed consent [1,3,5,6,7,8]. Although malpractice litigation in orthopedic surgery has been widely examined internationally, existing studies predominantly focus on insurance claims, first-instance court decisions, or disciplinary board outcomes. In particular, large U.S. claims-based analyses, most prominently those conducted by Daniels and colleagues in spine surgery, trauma-related orthopedics, and arthroscopic procedures, have provided detailed insights into allegation patterns, procedural risks, and litigation dynamics, yet remain largely confined to insurance and database-driven frameworks [9,10,11,12]. Recent multicenter and institutional litigation studies from Europe further support that both elective and trauma-related pathways generate recurrent medico-legal themes, but with jurisdiction-specific differences in complaint dynamics and outcomes [13]. Similarly, contemporary trauma-orthopedics–focused lawsuit series (2010–2021) highlight persistent patterns in allegations and outcomes, reinforcing the need for high-level, structured comparisons [14].

In Türkiye, available studies have mainly addressed survey-based perceptions of malpractice risk among orthopedic surgeons [15] or have focused on specific clinical contexts such as emergency department–related negligent homicide [16]. While these investigations provide valuable insights into physicians’ experiences and selected medico-legal scenarios, they do not evaluate finalized judicial interpretations derived from Supreme Court (Yargıtay) decisions.

High-court decisions are uniquely valuable because they represent the final stage of judicial review, offering authoritative interpretations of causality, fault attribution, and standards of care. High-court–level reasoning is particularly important in malpractice research because it often resolves disputed standards-of-care and causality questions that remain ambiguous in lower-court or claim-only datasets [2,13]. Consequently, systematic analyses based on Supreme Court decisions involving orthopedic specialists remain scarce in the Turkish literature, limiting direct comprehensive comparison with international high-level medico-legal data.

Therefore, the primary objective of this study is to analyze orthopedic malpractice cases adjudicated by the Turkish Supreme Court over a 15-year period (2010–2025), in order to identify recurrent allegations, judicial outcomes, and determinants of fault attribution. A secondary objective is to compare national findings with international data to contextualize Türkiye’s medico-legal patterns within the global orthopedic malpractice literature.

## 2. Materials and Methods

### 2.1. Study Design

This research was conducted as a retrospective, descriptive, and observational study analyzing orthopedic medical malpractice cases adjudicated by the Turkish Court of Cassation (Yargıtay). All publicly accessible high-court criminal and civil decisions involving orthopedic specialists between 1 January 2010 and 1 November 2025 were included, covering a continuous 15-year national dataset. The purpose of this design was to characterize litigation patterns, identify determinants of fault attribution, and evaluate judicial reasoning trends in orthopedic malpractice claims.

No human or animal subjects were contacted, and no clinical interventions were performed; thus, ethical committee approval was not required, as the dataset is entirely composed of publicly accessible legal documents.

The study period (2010–2025) was deliberately selected to ensure a sufficiently long and uninterrupted timeframe, allowing identification of stable litigation patterns and temporal trends in high-court malpractice decisions. During this period, no major regulatory changes affecting public access to Supreme Court decisions or the fundamental structure of medical liability adjudication were identified.

### 2.2. Legal Framework of Medical Malpractice Litigation in Türkiye

In Türkiye, medical malpractice claims may proceed through both criminal and civil judicial pathways, depending on the nature of the alleged act and its consequences. Criminal proceedings typically involve allegations such as negligent injury or negligent homicide, whereas civil proceedings primarily address compensation claims arising from alleged professional fault.

Judicial decisions rendered by first-instance courts may be reviewed by regional appellate courts and ultimately by the Supreme Court of Cassation (Yargıtay), which represents the highest judicial authority for both criminal and civil cases. In this manuscript, the terms “Supreme Court” and “Court of Cassation” are used interchangeably and refer exclusively to Yargıtay.

During adjudication, courts frequently rely on expert medical opinions to assess causality, adherence to standards of care, and fault attribution. These expert evaluations are most commonly provided by the Forensic Medicine Institute or the Higher Health Council. Importantly, these institutions function as independent expert advisory bodies supplying scientific and technical opinions to the judiciary, but they do not issue binding judicial decisions.

Because Supreme Court decisions represent finalized legal interpretations, the inclusion of both criminal and civil cases in the present analysis allows a broader evaluation of judicial reasoning and fault attribution in orthopedic malpractice litigation, independent of procedural differences in evidentiary thresholds.

### 2.3. Data Source and Case Identification

All data were obtained from the official website of the Turkish Supreme Court (https://karararama.yargitay.gov.tr/, accessed on 1 November 2025), which provides unrestricted public access to approximately nine million judicial decisions. No login, registration, or special permissions are required to view these documents.

A structured electronic search was conducted using the following keywords—selected based on relevance to orthopedic practice and medico-legal terminology: “orthopedics”, “orthopedic specialist”, “fracture management”, “surgical error”, “negligent injury”, “negligent homicide”, “medical malpractice”.

All retrieved records were manually screened by two independent reviewers to ensure accuracy and reduce selection bias. Cases were included if an orthopedic surgeon was directly named as the defendant or indirectly involved through consultation, follow-up, or shared surgical care.

Cases unrelated to orthopedic practice, duplicate entries, or incomplete records lacking sufficient legal detail were excluded.

### 2.4. Data Extraction and Variables

A standardized data extraction template was developed to ensure reproducibility of the analysis. The following variables were collected from each decision:

1. Demographic and Legal Variables
  - High-court chamber (criminal or civil)
  - Year of decision
  - Appeal status (affirmed, overturned)
2. Clinical and Case-Related Variables
  - Type of alleged crime: negligent injury, negligent homicide, misconduct, failure of duty, forgery, etc.
  - Case category: primary (directly targeting the orthopedic specialist) vs. secondary (orthopedic involvement resulting from emergency or multi-specialty care).
  - Clinical context:
    − Trauma management
    − Elective orthopedic surgery
    − Postoperative complication
    − Diagnostic delay or failure
  - Initial court outcome: acquittal or conviction
    − For the purposes of this study, primary liability refers to cases in which the orthopedic specialist was the principal defendant and directly responsible for the alleged malpractice. Secondary liability denotes cases in which orthopedic involvement occurred indirectly—such as through consultation, shared care, or multidisciplinary emergency management—and liability assessment depended on contributory responsibility rather than primary clinical decision-making.
3. Judicial Reasoning and Fault Assessment
  Fault attribution by the high court:
    − Physician at fault;
    − Physician not at fault;
    − Additional expert evaluation required (e.g., Forensic Medicine Institute, Higher Health Council).
  Legal arguments cited by the court:
    − Causal link analysis;
    − Adherence to medical standards;
    − Adequacy of informed consent;
    − Documentation quality;
    − Evaluation of postoperative care;
    − Technical considerations regarding surgical procedures.

All data were independently cross-checked to ensure consistency.

### 2.5. Data Availability Statement

Because this research relies solely on publicly available records, the complete dataset—including case numbers, decision years, and extracted variable matrix—can be provided to readers upon reasonable request.

All judicial documents are accessible at: https://karararama.yargitay.gov.tr.

No restrictions apply to their use.

## 3. Results

A total of 71 Supreme Court decisions involving orthopedic specialists were identified through a comprehensive review of high-court archives covering the last 15 years as of 1 November 2025. The most common alleged offense was negligent injury, reported in 54.9% of cases, followed by various forms of negligent homicide (17%). Additional accusations included misconduct (8.4%), neglect of duty (8.4%), and rare instances of forgery or combined offenses.

Regarding first-instance court decisions, after reclassification of categories, 36 cases (50.7%) resulted in acquittal, including those in which the outcome was deemed a medical complication. Convictions—including criminal judgments and compensation-related rulings—were identified in 35 cases (49.3%). The detailed distribution of these outcomes is shown in Table 1.

In the appellate phase, the Supreme Court affirmed 38 decisions (53.5%) and overturned 33 decisions (46.5%). Most reversals were attributed to incomplete medical evaluation, insufficient causality analysis, or the need for additional expert reports from the Forensic Medicine Institute or High Health Council. These appellate outcomes are summarized in Table 2.

Evaluation of fault attribution demonstrated that orthopedic physicians were found not at fault in 29 cases (40.8%) and at fault in 21 cases (29.6%). In an additional 21 cases (29.6%), the Court ordered further expert review before a final judgment could be reached. Fault categories are detailed in Table 2.

With respect to case characteristics, primary claims directly targeting orthopedic specialists constituted the majority of files (78.8%), while 19.7% involved secondary or indirect orthopedic responsibility. One case (1.4%) did not specify the nature of involvement. These distributions are also displayed in Table 2.

Overall, orthopedic malpractice claims in Türkiye predominantly centered on allegations of negligent harm, with trauma-related management serving as a recurring theme. Nearly half of initial acquittals underwent additional appellate evaluation, highlighting the central role of causality, adherence to orthopedic standards of care, informed consent quality, and medical documentation adequacy in judicial assessments.

## 4. Discussion

The present study represents the most comprehensive evaluation of 15 years of Turkish high-court malpractice litigation involving orthopedic specialists, offering crucial insights into national medicolegal patterns and enabling meaningful comparison with international data.

### 4.1. Patterns of Alleged Offenses and Case Characteristics

In Türkiye, negligent injury was the most frequent allegation in orthopedic malpractice litigation, followed by negligent homicide and professional misconduct. This overall profile is broadly consistent with international datasets indicating that orthopedic claims most commonly arise from trauma-related care pathways and are frequently linked to diagnostic delay, perioperative management issues, and postoperative complications [1,3,5,6,7,8]. Large national claims analyses from the United Kingdom demonstrate that delayed or failed treatment and surgical error constitute leading causes of orthopedic litigation, emphasizing the central role of time-sensitive decision-making and procedural risk in this specialty [2,4].

In our dataset, most cases were primary claims directly targeting orthopedic specialists, suggesting that litigation predominantly focuses on the surgeon’s own clinical decision-making and management responsibilities rather than solely shared or multidisciplinary care. International medico-legal series similarly report that orthopedic litigation often concentrates on surgeon-directed allegations, with recurring themes including missed fractures, delayed diagnosis, and postoperative adverse outcomes [1,3,6,7,8]. Consistent with our findings, European and UK-based trauma and orthopedic litigation studies report that claim patterns repeatedly cluster around injury management, delays in diagnosis or treatment, and perioperative care deficiencies [4,13].

Although the proportion of trauma-related cases varies between healthcare systems, institutional and multicenter European datasets indicate that both emergency trauma care and elective orthopedic procedures may generate malpractice claims; nevertheless, diagnostic error and perioperative/postoperative management failures remain the dominant allegation domains across settings [13,14].

Recent trauma-focused lawsuit series covering the period 2010–2021 further demonstrate that allegations of diagnostic delay, inadequate management, and communication or informed-consent deficiencies are recurrent features of orthopedic litigation, closely aligning with the thematic distribution observed in our Supreme Court sample [14].

### 4.2. Judicial Outcomes and Fault Attribution

Our analysis revealed that 50.7% of initial court decisions resulted in acquittal, aligning with international observations that most orthopedic malpractice claims do not ultimately result in physician liability [1,6,8]. Similar acquittal-dominant outcome distributions have been described in large national and institutional orthopedic litigation datasets from the USA and Europe, including claims-based analyses reported by Daniels and colleagues, where high claim volumes contrast with relatively low final liability rates [2,6,9,10,11,12].

Following Supreme Court review, 53.5% of decisions were affirmed and 46.5% were overturned, most commonly due to insufficient causality analysis or incomplete expert evaluation. High appellate reversal rates associated with deficiencies in causality assessment and expert reporting have also been documented in national-level medico-legal analyses, particularly in systems that rely heavily on post hoc expert appraisal [2]. These findings suggest that appellate scrutiny serves as a corrective mechanism rather than an indicator of inconsistent judicial practice.

The relatively high rate of overturned decisions underscores the central role of expert medical assessment in high-court malpractice adjudication. Similar reliance on expert appraisal mechanisms has been reported in other legal systems, including France [3] and China [5]. Across jurisdictions, expert reassessment appears to be a key determinant in distinguishing unavoidable complications from true deviations from accepted standards of care, especially in technically complex orthopedic and trauma-related cases [3].

Physicians were ultimately found not at fault in 40.8% of cases, while fault was upheld in 29.6%—rates comparable to global conviction patterns reported in the orthopedic malpractice literature [1,3,8]. European insurance-based and court-level analyses further indicate that confirmed physician fault typically remains below one-third of claims, despite prolonged legal proceedings and multiple stages of judicial review [6]. In the remaining cases, additional expert review was required prior to final adjudication, highlighting the inherent complexity of orthopedic medicolegal assessments and the challenges associated with establishing causal linkage and evaluating technical performance [3].

### 4.3. Complications vs. Negligence

Several overturned decisions involved misclassification of postoperative complications as negligence at the first-instance level. This distinction is critical in orthopedic surgery, where adverse outcomes may occur despite adherence to accepted standards of care.

International studies similarly emphasize the importance of differentiating unavoidable complications from true malpractice [3,7,17]. Our findings demonstrate that high-court review plays a corrective role in refining this distinction, often reclassifying complication-related cases as non-negligent when causality or breach of standard cannot be conclusively established. Comparable corrective roles of appellate or high-level judicial review have been described in European orthopedic malpractice analyses, where higher courts frequently overturn lower-court decisions that inadequately distinguish between unavoidable complications and professional fault [3,6]. Similar distinctions between surgical complications and true malpractice have also been emphasized in large U.S. claims-based orthopedic litigation studies reported by Daniels and colleagues. In spine surgery and trauma-related orthopedic procedures, adverse postoperative events—including neurological deterioration or the need for reoperation—are frequently interpreted as inherent procedural risks rather than deviations from accepted standards of care, supporting the reclassification of complication-related claims as non-negligent in the absence of clear diagnostic or technical error [9,10,11,12].

The complexity of orthopedic surgery, particularly in trauma and high-risk procedures, further complicates the medico-legal assessment of complications. Surgical site infections, delayed union, implant failure, and neurovascular injury may arise even under optimal care conditions. International medico-legal literature consistently underlines that adverse outcomes alone are insufficient to establish negligence in the absence of demonstrable deviation from accepted standards of care and a clearly defined causal link, reinforcing the need for expert reassessment in orthopedic litigation [3,6,7,18].

### 4.4. Psychological Impact, Burnout, and Defensive Medicine

Turkish survey evidence shows a high prevalence of burnout, defensive medical behavior, and emotional distress among orthopedic surgeons facing malpractice claims [16]. These findings are consistent with U.S. and European data [7,8,18], suggesting that litigation threatens not only physicians’ financial and professional integrity but also their mental well-being. High-risk subspecialties such as arthroplasty, spine, and hand surgery have been frequently associated with malpractice complaints in international series [1,3,7,8].

Although the present study did not directly assess psychological outcomes, the frequent recurrence of prolonged judicial processes and appeals observed in our dataset suggests a potential indirect contribution to defensive medical practices, as also described in the broader literature [19,20]. Similarly, large U.S. claims-based orthopedic malpractice analyses reported by Daniels and colleagues highlight that sustained litigation exposure and high claim volumes may contribute to defensive medical practices among surgeons, reinforcing the broader medico-legal impact of malpractice beyond individual case outcomes [9,10,11,12].

### 4.5. International Context and Converging Global Themes

Despite differences in healthcare organization and legal frameworks, several recurring themes consistently emerge across countries in orthopedic malpractice litigation. These include an increasing volume of claims, trauma-related care as a leading trigger for litigation, frequent allegations of diagnostic delay, and relatively low final conviction rates following judicial review [1,2,3,5,6,7,8].

The findings of the present study demonstrate substantial alignment between Turkish Supreme Court decisions and international orthopedic malpractice patterns. In particular, the predominance of trauma-related cases, the central role of causality assessment, and the frequent re-evaluation of first-instance judgments by higher courts reflect common global medicolegal challenges. This convergence suggests that orthopedic malpractice litigation is shaped by universal clinical and legal dynamics rather than country-specific factors alone.

### 4.6. Implications for Clinical Practice and Risk Management

Based on judicial patterns observed in Turkish Supreme Court decisions and contextualized through international literature, several practice-related implications can be inferred. These implications should be interpreted as contextually derived considerations informed primarily by judicial reasoning and supported by existing evidence, rather than direct empirical outcomes measured within the present study.

1. Strengthen Documentation Practices

High-court judgments frequently emphasize inadequate or inconsistent medical documentation as a critical weakness in malpractice adjudication. Judicial reasoning often relies on operative notes, informed consent records, and follow-up documentation when assessing causality and standard-of-care compliance. These findings suggest that comprehensive and consistent documentation may play an important role in reducing medicolegal ambiguity. International malpractice analyses similarly identify documentation quality as a central determinant in fault attribution and appellate decision-making, particularly in surgical specialties with complex care pathways [13].

2. Enhancing the Quality of Informed Consent

Failure to provide detailed, procedure-specific informed consent remains a recurrent issue in malpractice litigation. Judicial evaluations highlight the importance of clear communication and documentation of patient understanding. Although the present study did not assess consent practices directly, existing evidence suggests that structured and standardized consent processes may reduce misunderstandings and subsequent legal disputes. Prior medico-legal studies indicate that deficiencies in informed consent documentation are frequently cited in malpractice claims and may independently contribute to adverse legal outcomes even in the absence of technical error [3].

3. Improve Diagnostic Vigilance and Early Recognition

Missed fractures, delayed diagnosis of compartment syndrome, deep infections, and postoperative deterioration are frequently cited in malpractice claims in both Türkiye and international series [1,3,5,16,21]. Judicial decisions repeatedly emphasize diagnostic timing and reassessment, suggesting that structured evaluation protocols and timely multidisciplinary input may mitigate diagnostic failure in high-risk clinical scenarios. Comparable diagnostic-risk patterns have been consistently reported in international orthopedic litigation studies, reinforcing the global relevance of early recognition and reassessment in reducing malpractice exposure [7].

4. Formalize Postoperative Follow-Up Protocols

Many adverse outcomes addressed in high-court decisions relate to insufficient postoperative monitoring and follow-up. Judicial reasoning frequently refers to discharge planning, continuity of care, and early recognition of complications. These observations suggest that structured follow-up strategies may represent a relevant component of risk reduction in orthopedic practice. International medico-legal literature supports that failures in postoperative surveillance and follow-up communication are common contributors to litigation in orthopedic surgery [6,13].

5. Promoting Medicolegal Awareness During Residency Training

Although the present study did not evaluate residency curricula, international literature consistently emphasizes the importance of medicolegal education, including documentation standards, communication skills, and legal responsibilities [7,16,17,21]. Increased awareness of medicolegal principles during training may support improved risk management in clinical practice.

6. Establish Institutional Malpractice Support Systems

Hospitals and healthcare institutions play a key role in managing medicolegal risk. Previous studies have demonstrated that institutional support mechanisms—such as legal advisory units and structured case review processes—may help reduce psychological burden and defensive medical behavior among physicians [19,20]. Judicial patterns suggesting prolonged litigation and repeated appeals further underscore the potential value of institutional support.

7. Developing National Orthopedic Malpractice Registries

Standardized reporting systems have been shown to facilitate the identification of high-risk interventions, enable outcome monitoring, and support quality-improvement initiatives across medical disciplines [22,23]. Within the context of orthopedic malpractice litigation, national registries may provide valuable aggregate data to inform policy development and preventive strategies.

8. Facilitate Multidisciplinary Decision-Making in High-Risk Cases

Complex trauma cases, spine surgery, and limb-salvage procedures frequently involve multiple specialties and high medicolegal risk. Judicial evaluations often highlight shared responsibility and communication among care teams, suggesting that multidisciplinary decision-making may contribute to improved patient safety and reduced individual medicolegal burden. International studies examining orthopedic litigation in complex care settings similarly emphasize communication failures and fragmented decision-making as recurrent contributors to adverse medico-legal outcomes [6].

These recommendations may contribute to reducing malpractice frequency, enhancing patient satisfaction, and improving clinical outcomes.

### 4.7. Limitations

Several limitations should be acknowledged when interpreting the findings of this study. First, the analysis is restricted to publicly accessible Supreme Court decisions, which represent only the final stage of the judicial process. Cases resolved at lower court levels, settled privately, or dismissed without appeal were not captured.

Second, some judicial decisions lacked detailed clinical information, limiting in-depth medical interpretation. Third, expert opinions cited within decisions were occasionally incomplete or inconsistently reported, potentially affecting uniform comparison across cases. In addition, consistently reported data regarding the duration of legal proceedings were not available, precluding reliable time-to-resolution analyses.

Finally, international comparisons were derived from published literature, and differences in legal systems, compensation models, and reporting practices may limit direct comparability. Nevertheless, the study provides a comprehensive overview of high-court orthopedic malpractice litigation and offers valuable insight into national and international medicolegal patterns.

## 5. Conclusions

This 15-year analysis of Turkish Supreme Court decisions provides a comprehensive overview of orthopedic malpractice litigation in Türkiye. Negligent injury remains the most frequent allegation, with trauma-related care, diagnostic delay, and postoperative complications representing the leading triggers for litigation. Despite the high number of claims, final conviction rates remain relatively low, reflecting the judiciary’s emphasis on causality assessment and expert medical evaluation.

The high proportion of overturned decisions underscores the critical role of expert opinion, thorough documentation, and adherence to accepted standards of care in medicolegal adjudication. The observed alignment between Turkish and international malpractice patterns suggests that orthopedic litigation is driven by shared global challenges.

Overall, this study contributes high-level judicial evidence to the orthopedic literature and may help inform future risk-management strategies, medicolegal education, and policy initiatives aimed at improving patient safety while reducing the burden of malpractice litigation.

## Figures and Tables

**Table 1:** Distribution of Initial Court Decisions.

Initial Court Decision Category	*n* (%)
Acquittal (including cases classified as medical complication)	36 (50.7%)
Conviction (including criminal convictions and compensation-related judgments)	35 (49.3%)
Total	71 (100%)

**Table 2:** Supreme Court Outcomes, Fault Attribution, and Case Characteristics.

Variable	Categories	*n* (%)
**Cassation Outcome**	Affirmed	38 (53.5%)
	Overturned	33 (46.5%)
**Fault Attribution**	Physician at fault	21 (29.6%)
	Physician faultless	29 (40.8%)
	Additional expert report requested	21 (29.6%)
**Case Type**	Primary claim	56 (78.8%)
	Secondary involvement	14 (19.7%)
	Not specified	1 (1.4%)

## Data Availability

All data analyzed in this study are publicly available in the Turkish Supreme Court judicial database (https://karararama.yargitay.gov.tr). Additional extracted datasets created during the analysis can be provided by the authors upon reasonable request.
